# Annotations

**Published:** 1899-05-06

**Authors:** 


### ANNOTATIONS.

### A Fly-proof House.

Lady Priestley has done good service in giving literary form and shape to certain well known facts as to the *rôle* played by winged creatures in the dissemination of disease. This she has done in the *Nineteenth Century*, where she has summarised the observations which have been recently accumulated in regard to "winged carriers of disease." She starts with the demonstration made by Mr. Burgess at the Royal Society two or three years ago, when he showed how common house-flies which had placed their feet on a culture of the *Bacillus prodigiosus* set up a fresh growth of these bacilli when made to walk over sliced potatos, even though in the interval they had been allowed to roam at liberty in a large room for several hours. "In the natural course of time he had the satisfaction of seeing a perfect garden of the *Bacillus prodigiosus* spring up wherever their feet had touched." Then comes the analogous experiment with the diphtheria bacillus, and the observations showing the several ways in which the infection of cholera may be spread by flies. Lastly, Lady Priestley tells the wonderful story of the malaria parasite and of the *filaria nocturna*, and points to cattle tick and the Tsetse-fly as illustrations of the roundabout manner in which infection is sometimes spread. It is an interesting article and one well calculated to show to the lay mind some of the intricacies of the problems of disease. Still we are afraid that cholera, malaria, sleeping sickness, and the whole host of "tropical diseases" which have stirred Lady Priestley to enthusiasm are a long way off and quite impersonal to the average Englishman. It is well then to insist that, although details differ, the principles of medicine and the principles of biology are the same all the world over, and that "winged carriers of disease" are potent factors—far more potent factors than some think—in the dissemination of various infections. The "aerial diffusion of infection" has always been a most unsatisfactory expression. But if we recognise that the air contains many living things and that the germs of small-pox are certainly as easy to carry as is the *Bacillus prodigiosus*, not only does an explanation of much that is unexplained by current phraseology seem near at hand, but also a means of prophylaxis. Lady Priestley's discription of the "fly-proof" house in which dwelt her host, "the most agreeable and witty man of his day, the distinguished physician, novelist, and poet, Oliver Wendell Holmes," is full of suggestiveness in regard to hospital construction.

### Fleeing from Disease.

There are some diseases the nature of which is so obscure, the treatment so doubtful, and the outcome so serious, that in their presence the better part is to run away, and of such diseases plague seems to be the very type. Moreover, the very reasons which we have for believing it to be a house disease—one dependent perhaps on rats, perhaps on fleas, or other parasites of the dwelling, but at any rate upon the sum total which we call the house—are reasons also for running away when such a malady breaks out. And if we are to judge by the evidence given before the Plague Commission the evacuation of the villages attacked, and even of towns of considerable size in which epidemics have occurred, has been by far the most successful method of dealing with such outbreaks; that is, if combined with disinfection and measures for ensuring the cleansing of the houses and their exposure to light and air. What is still more interesting is that it was observed in the Punjab, that even when plague happened to be reintroduced into a village which had been evacuated and disinfected, it showed little tendency to spread, so that the evacuation of a few houses was sufficient to check the second outbreak. This seems to give the clue, not perhaps to the *causa causans*, as it has been called, which we must admit to be a bacillus, but to those contributory causes which are at least so important as to be essential to the epidemic prevalence of the disease; namely, lack of air and light, and the presence of dirt and vermin. Clear these away and even in the presence of infection the outbreak dies down. It has been held by some that this method of dealing with epidemics by evacuation, useful as it is shown to be in the case of villages and small communities, is quite out of the question in large towns. It is interesting then to observe that even in towns of considerable size the method has been adopted with marked success. In Baroda for example, a town of 112,000 inhabitants, at least 40,000 people removed themselves into open spaces outside the city limits, while the city proper was entirely evacuated, and large numbers were afterwards removed into the camp. This was done in March and only nine cases occurred subsequently in the camps, and the whole epidemic ceased in the course of April. In the second outbreak in Karachi the whole of the town proper was evacuated, and with successful results, although the people were allowed to return to their houses in the day time. Whole quarters of the town with an aggregate population of 40,000 were completely evacuated, and it was shown that evacuation for a couple of months, together with the adoption of measures for ensuring the access of light and air, such as removing the roofs of houses where this was found necessary, ensured safety from recurrence on the return of the inhabitants to their homes.

### Consumptive Patients at Natal.

The *Times* paper recently contained a letter from Sir Walter Peace, the Agent-General for Natal, conveying what seems to be a much-needed warning against the "growing practice," among medical men in this country, "of recommending consumptive patients to go to Natal and other parts of South Africa." Sir Walter declares that, under certain conditions, the advice is good; but points out that it has often been acted upon with lamentable consequences. If the disease is only threatened, or only slightly developed, and there is reasonable prospect of recovery, patients who have sufficient means to support themselves in comfort may go to South Africa with advantage. But there is no provision for the treatment of consumptives otherwise than in the hospitals, the expenses of sickness are greatly in excess of what they are in England, and the private benevolence of the small European community is already overtaxed. There is too much fear of a recurrence of what was once a common practice, that of sending patients from home only to die; a practice which, fifty years ago, filled Madeira and other supposed "health resorts" with English graves, and which, while it increased the sufferings of the dying, increased also the distress of their relatives. Sir Walter writes by the express direction of his Government, and it would be impossible to give too much publicity to his caution.

